# Proceedings of the 15th International Congress on Circumpolar Health

**DOI:** 10.3402/ijch.v72i0.22447

**Published:** 2013-08-05

## Abstract

Proceedings of the 15th International Congress on Circumpolar Health August 5-10, 2012, Fairbanks, Alaska, USA. This extensive publication includes nearly 100 full length papers, 90 extended abstracts and nearly 100 short abstracts. The full publication is freely available through the journal website.

